# Construction of a tunable multi-enzyme-coordinate expression system for biosynthesis of chiral drug intermediates

**DOI:** 10.1038/srep30462

**Published:** 2016-07-26

**Authors:** Wei Jiang, Baishan Fang

**Affiliations:** 1 Department of Chemical and Biochemical Engineering, College of Chemistry and Chemical Engineering, Xiamen University, Xiamen, 361005, China; 2 The Key Lab for Synthetic Biotechnology of Xiamen City, Xiamen University, Xiamen 361005, China; 3 The Key Laboratory for Chemical Biology of Fujian Province, Xiamen University, Xiamen, Fujian, 361005, China

## Abstract

Systems that can regulate and coordinate the expression of multiple enzymes for metabolic regulation and synthesis of important drug intermediates are poorly explored. In this work, a strategy for constructing a tunable multi-enzyme-coordinate expression system for biosynthesis of chiral drug intermediates was developed and evaluated by connecting protein-protein expressions, regulating the strength of ribosome binding sites (RBS) and detecting the system capacity for producing chiral amino acid. Results demonstrated that the dual-enzyme system had good enantioselectivity, low cost, high stability, high conversion rate and approximately 100% substrate conversion. This study has paved a new way of exploring metabolic mechanism of functional genes and engineering whole cell-catalysts for synthesis of chiral α-hydroxy acids or chiral amino acids.

Co-expression of multienzymes to construct an artificial catalysis system in *E. coli* has become a powerful tool to generate highly effective cell catalysts^1^,^2^,^3^,^4^,^5^,^6^,^7^,^8^,^9^,^10^,^11^, but little information is available about the coordinated expression of the multiple enzymes. It is reported that the optimal bioconversion by multi-enzyme catalysis occurs only when the individual catalytic steps are balanced^4^,^10^.

In organic synthesis, amino acids are widely used as chiral starting materials, catalysts and auxiliaries^12^. The unnatural amino acids, e.g. l-*tert*-leucine (l-Tle) as a component of the drugs Reyataz (HIV) and Telaprevir (HCV), play an important role in the food, cosmetic, agrochemical and pharmaceutical industries as pharmaceutically active peptides, pesticides and chiral ligands^10^,^13^,^14^. Moreover, many ligands of chemo-catalysts for asymmetric catalysis are constructed based on l-Tle^15^,^16^. These reports suggest the great potential of the development of the processes to synthesize l-Tle^17^. To date, various chemical methods for the synthesis of l-Tle and its derivatives have been explored^17^. However, biocatalysis usually offers more benefits than chemical methods, because the latter has the problems such as a complex process, low chiral selectivity, low production rate and environmental pollution. For biocatalysis, a cofactor-dependent route was implemented by using two enzymes, a formate dehydrogenase (FDH; EC 1.2.1.2) for coenzyme NADH regeneration *in situ* and a leucine dehydrogenase (LeuDH; EC 1.4.1.9) for reductive amination of trimethylpyruvate^10^. Cofactor regeneration is very important for the cofactor-dependent route due to the high cost of the used cofactor^18^.

Recently, to facilitate the biosynthesis of l-Tle, various strategies have been developed for constructing a coenzyme regeneration system. The enzymatic method, which depends on the combined use of purified and isolated enzymes^19^,^20^,^21^,^22^,^23^, has achieved high conversion and enantioselectivity, but it needs cofactor NADH recycling or isolation and the use of costly enzymes for added cofactor. Anne Menzel *et al.* have developed a whole-cell catalyst, bearing a LeuDH and a FDH as a “designer bug”-whole cell catalyst, resulting in high conversion^10^,^24^. However, this method shows a large gap in the enzyme activities of both enzymes by a factor >6.7 and it consumes double antibiotics (as the method employs two plasmids to express the LeuDH and FDH, respectively). To address these issues, we need to construct a straightforward and improved route. Specifically, the route should not only enable the direct use of a whole cell catalyst, containing both desired enzymes in a plasmid form, and the use of only the intracellular cofactor, but also should contain a regulatory expression system to coordinate the multiple enzymes. Synthetic biology, which demonstrates its broad application perspective in the fields of medicine, chemical synthesis and the production of energy, can be used to solve these problems.

In this work, we report the preparation of an economical and efficient catalyst for the synthesis of L-Tle, which is an important chiral amino acid. We attempted to build a regulatory expression system of LeuDH and FDH using series connection of protein-protein expressions and different strength RBS. To carry out this cascade in a designer cell catalyst, fine adjustment of the expression levels of the enzymes was required. We achieved this by varying the RBS to regulate the expression of the LeuDH and equip the FDH with the strongest RBS due to its known low enzyme activity^25^ (Fig. 1, for the detailed building process, refer to the experimental method and Fig. S1 in the Supporting information). Using a very strong RBS to regulate the expression level of FDH was also important for improving the efficiency of the NADH recycling of the cell catalysts^26^. We constructed three types of circuits with different RBS, B0034, B0030 and B0032 for exploring the optimal proportion of the coordinated expression of the FDH and LeuDH (B0034 is stronger than B0030, and B0032 is the weakest.). To this end, the BioBrick B0034 + FDH, B0034 + LeuDH, B0030 + LeuDH and B0032 + LeuDH for separate expression of genes and the BioBrick B0034 +  + LeuDH-B0034 + FDH (34L-34F), B0032 + LeuDH-B0034 + FDH (32L-34F) and B0030 + LeuDH-B0034 + FDH (30L-34F) for cascade expression of genes were constructed (Fig. 1 and Fig. S2).

The separate and cascade genes were expressed under the optimal conditions (Optimization procedure and the results are shown in the experimental method and Fig. S3 in the Supporting information). It was demonstrated that the circuits were successfully expressed by detecting the protein expression and enzyme activity for each circuit (Fig. 2 and Fig. S3). Then, these circuits as biocatalysts were employed for catalytic synthesis of L-Tle to determine the function of these circuits and detect the optimal control range of RBS. After the expression, the cells of 34L-34F (mix B0034 + FDH with B0034 + LeuDH as control), 32L-34F (mix B0034 + FDH with B0032 + LeuDH as control) and 30L-34F (mix B0034 + FDH with B0030 + LeuDH as control) were employed to synthesize Tle from trimethylpyruvic acid (TMA).

All *E. coli*/biobricks catalysts were employed to transform 100 mM TMA to the corresponding L-Tle (Fig. 3) and the target product was acquired in a highly optically pure form (>99% e.e., no D-Tle was detected by chiral HPLC analysis, so the e.e. value was considered to be greater than 99%). More than 78% of the substrate (TMA) was converted within 1 h (Fig. 3). The 30L-34F showed a good balance between the reduction and oxidation step because the amount of keto acid, TMA, was kept below 1% at the 4^th^ hour. However, 32L-34F and 34L-34F had surplus substrate in the reaction at the early stage, suggesting that the activity of the LeuDH was too high or too low without entering the reduction step. This phenomenon indicated that the coordination of enzymes’ activities in synthetic cascade routes is very important to end the rate-limiting steps^27^ and enhance product yield. Based on these results, the best RBS regulation interval was obtained.

The unbalance between the reduction and oxidation reactions for 34L-34F or 32L-34F can be ascribed to the excessive or insufficient activity of LeuDH in these systems (Fig. 3a,b). This hypothesis was confirmed by the appropriate activity of LeuDH in the 30L-34F, which substantially optimized the balance between the reduction and oxidation steps. Additionally, the 30L-34F had the highest conversion rate for the synthesis of L-Tle (Fig. 3d). The remnant of TMA was detected in the first few hours when the reduction step was inefficient (Fig. 3b), and the overall rate was limited by the reduction step. On the other hand, the remnant of TMA was also detected when the reduction reaction was stronger than the oxidation just like in 34L-34F (Fig. 3a). Here, the overall efficiency was determined by the oxidation step. Additionally, the conversion rate of all the three cascade enzyme systems was higher than that of the three separate enzyme systems (Fig. 3a–c). Double enzyme expression within one cell and immediate mixing would form the substrate channel^27^,^28^,^29^,^30^ and enhance the transfer efficiency.

Previous enzymatic syntheses of Tle include using a branched chain aminotransferase coupled with glutamate dehydrogenase (GDH)/FDH to produce L-Tle with a conversion rate of 90%^31^. The comparatively low substrate concentration does impede its potential in industrial applications. Based on the combined use of LeuDH and FDH, another reductive amination path for the synthesis of L-Tle was also constructed^32^. This efficient but cofactor-dependent path has been successfully implemented with separate FDH and LeuDH^22^,^33^,^34^, but it had the disadvantages of laborious isolation, enzyme purification and the generation of a weak stability of the enzymes under these processes. Additionally, the L-Tle produced exhibited high conversion and space-time yield (93% and 366 g L^−1^ d^−1^, respectively)^33^. Since the use of costly and isolated enzymes was found to be defective, a whole-cell catalyst containing a FDH from *C. boidinii* and a LeuDH from *B. cereus* was established and used in L-Tle production^10^,^24^,^35^. L-Tle was obtained with the whole-cell catalyst in >99% e.e. and 95% conversion, but it had a low space-time yield of 124 g L^−1^ d^−1 ^10. Meanwhile, the double plasmids required result in a doubling of the consumption of the antibiotics required for plasmid expression. With 40 g/L wet bacteria, a high space-time yield of 786 g L^−1^ d^−1^ was obtained by Weiming Liu *et al.*^24^. Compared with their results, the space-time yield was 247 g L^−1^ d^−1^ in the present work, but only 10 g/L wet bacteria was used, half of the antibiotic was consumed and only one plasmid was used to co-express the genes for the optimal L-Tle production. Therefore, the design idea in the regulatory expression system for the coordination of enzymes could be used to synthesize other crucial α-hydroxy acids, amino acids, and chiral drug intermediates^36^,^37^.

In conclusion, a regulatory expression system for the coordination of enzymes by cascade connection of protein-protein expressions and regulation of the strength of RBS was developed for the biosynthesis of L-Tle, an important chiral drug intermediate. Regulation of the expression of genes by RBS strength was executed to equilibrate the activity of the two enzymes and obtain the highest yield of the target product. Efficient biosynthesis of chiral drug intermediates has been demonstrated by employing freeze dried whole cell-catalyst with 100 mM substrate concentration, producing the target product L-Tle in high yield (approximately 100%) and enantiopure form (>99% e.e.) without the need for chromatographic purification. This work demonstrates that the regulatory expression of enzymes in synthetic cascade routes could be a very significant tool for exploring the metabolic mechanism of functional genes and engineering whole cell-catalysts and it can be used for highly efficient synthesis of other chiral drug intermediates.

## Methods

All the experimental procedures of the construction and expression of the genetic circuits (The seven circuits, B0034 + LeuDH, B0032 + LeuDH, B0030 + LeuDH, B0032 + FDH, B0030 + LeuDH-B0034 + FDH, B0032 + LeuDH-B0034 + FDH and B0030 + LeuDH-B0034 + FDH), enzyme and protein assays were described in detail in the Support information.

### Production of L-*tert*-leucine by using the genetic circuits

The cell of 34L-34F (mix B0034 + FDH with B0034 + LeuDH as control), 32L-34F (mix B0034 + FDH with B0032 + LeuDH as control) and 30L-34F (mix B0034 + FDH with B0030 + LeuDH as control), which were employed to synthesize Tle from trimethylpyruvic acid (TMA), were inoculated, induced, cultivated, collected, centrifuged (8000 rpm, 10 min), washed with phosphate buffer (0.2 M, pH 7.8) twice, collected and store the cells at −80 °C overnight. After that, the cells were thawed at 20 °C, re-suspended with NH_3_·H_2_O-NH_4_Cl buffer (1 M, pH 7.5), introduced into a 15 mL reaction systems and the cells were broken by Ultrasonic cell disruption. The reaction conditions (15 mL): TMA (100 mM), ammonium formate (200 mM), NADH (0.01 g/L) and cell-free extracts of *E. coli* cells (4 g/L wet cells) in NH_3_OH-NH_4_Cl buffer (1 M, pH 8.0), 37 °C, 220 rpm. The samples (500 μL) were collected at 1, 2, 4, 6, 8, 10, 12, 24, 48, 72, 96 h. The product was filtered via ion-exchange resin (Amberlite IR-120H) and characterized by high-performance liquid chromatography (HPLC). The detected conditions consisted of: Column: Chirex3126, Phenomenex; Eluent: 2% CuSO_4_ aqueous solution/isopropanol =  95/5, flow rate: 1.0 ml/min). The enantiomeric purity of the product was also determined. The conversion rate was defined as the mole concentration ratio of producing *tert*-leucine (The concentration of *tert*-leucine in the enzymatically synthesised product was detected by HPLC) and starting substrate concentration (The starting substrate concentration in the reaction system was determined by the experimental scheme, it was sure by weighing and then it was added into the reaction system before the start of the reaction). The e.e. of L-*tert*-leucine was calculated as follows: e.e. (%) = (A_1_ − A_2_)/(A_1_ + A_2_) × 100, A_1_ and A_2_ were the peak areas of L-*tert*-leucine and D-*tert*-leucine, respectively^24^. All chemical used in this work were analytical reagent or higher, obtained from Sigma (United States) or Sangon Biotech Co., Ltd. (Shanghai, China).

## Additional Information

**How to cite this article**: Jiang, W. and Fang, B. Construction of a tunable multi-enzyme-coordinate expression system for biosynthesis of chiral drug intermediates. *Sci. Rep.* **6**, 30462; doi: 10.1038/srep30462 (2016).

## Supplementary Material

Supplementary Information

## Figures and Tables

**Figure 1:**
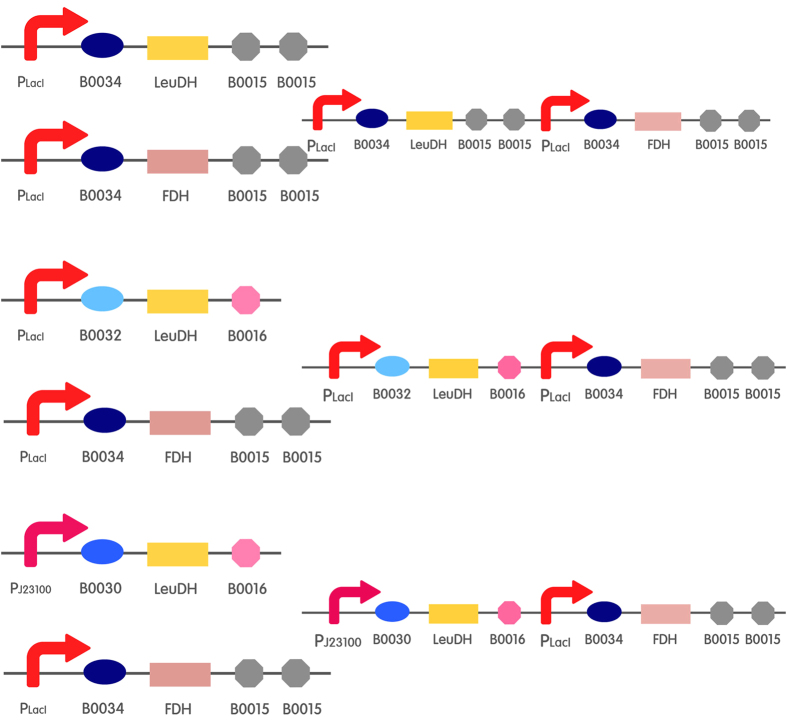
Overview of the flexible design of different expression constructs used in this work. Legend: LeuDH: leucine dehydrogenase; FDH: formate dehydrogenase; P: IPTG-inducible promoter; B0034, B0032 and B0030: RBS; B0015 and B0016: terminator.

**Figure 2:**
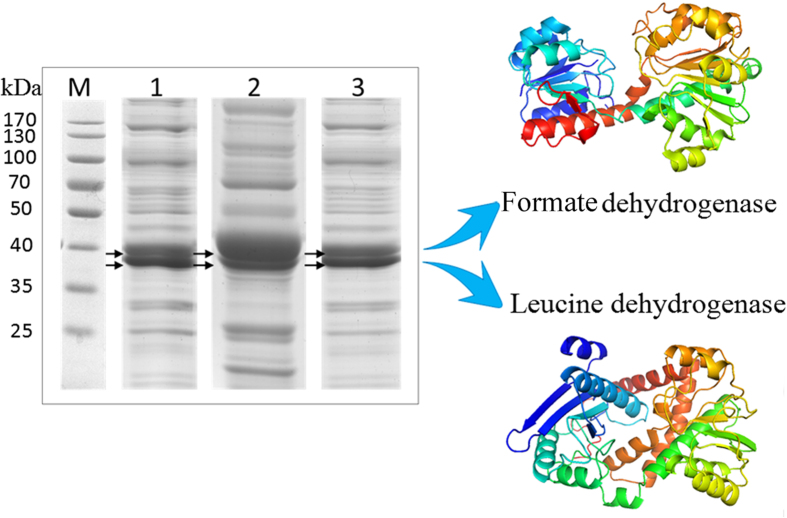
12% SDS-PAGE analysis of the protein expression of genetic circuits of 34L-34F, 32L-34F and 30L-34F. Lane M: Protein marker. Lane 1: recombinant bacterium (harboring 32L-34F) induced by IPTG. Lane 2: recombinant bacterium (harboring 34L-34F) induced by IPTG. Lane 1: recombinant bacterium (harboring 30L-34F) induced by IPTG.

**Figure 3:**
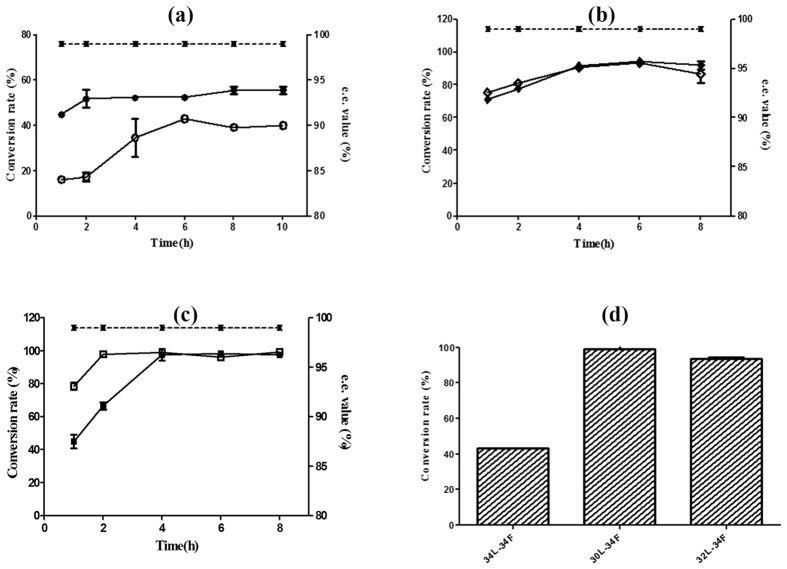
The conversion ratio of L-*tert*-leucine synthesis reaction catalyzed by the circuits. (**a**) 34L-34F (○, e.e value was shown as ▼), mixing B0034 + FDH with B0034 + LeuDH (●, e.e value was shown as ▲); (b) 32L-34F (○, e.e value was shown as ▼), mixing B0034 + FDH with B0032 + LeuDH (◆, e.e value was shown as ▲); (c) 30L-34F (□, e.e value was shown as ▼), mixing B0034 + FDH with B0030 + LeuDH (■, e.e value was shown as ▲); (**b**) The highest conversion ratio of L-*tert*-leucine synthesis reaction was catalyzed separately by the 34L-34F, 32L-34F and 30L-34F. Reaction conditions: TMA (100 mM), ammonium formate (200 mM), NADH (0.01 g/L) and cell-free extracts of *E. coli* cells (4 g/L wet cells) in NH_3_OH –NH_4_Cl buffer (1 M, pH 8.0), 37 °C, 220 rpm. All experiments were performed in triplicate and repeated three times. Error bars represent the standard deviation.
