# Supplementary material for: Construction of a tunable multi-enzyme-coordinate expression system for biosynthesis of chiral drug intermediates
Source: Sci Rep. 2016 Jul 26;6:30462. doi: 10.1038/srep30462 (PMC4960608; doi:10.1038/srep30462)
Supplement: Supplementary Information [file srep30462-s1.pdf]

Construction of a tunable multi-enzyme-coordinate expression system for biosynthesis of chiral drug intermediates

Wei Jiang[1,2], Baishan Fang[1,2,3※]

[1]Department of Chemical and Biochemical Engineering, College of Chemistry and Chemical Engineering, Xiamen University, Xiamen, 361005, China

[2]The Key Lab for Synthetic Biotechnology of Xiamen City, Xiamen University, Xiamen 361005, China

[3]The Key Laboratory for Chemical Biology of Fujian Province, Xiamen University, Xiamen, Fujian, 361005, China

Running title: A tunable multi-enzyme-coordinate expression system for biosynthesis of chiral drug intermediates

※Corresponding author:

Prof. Bai-Shan Fang

Tel: +86-0592-218-5869

Fax: +86-0592-218-4822

E-mail: fbs@xmu.edu.cn

Support information

Figure legends

Fig.S1. The methods and detail process of constructing the genetic circuits.

Fig.S2. The physical maps of the genetic circuits.

Fig.S3. The suitable concentration of IPTG for expression of the genetic circuits. (a) Effect of IPTG on the enzyme expression of the B0034+FDH. The enzymes were induced by different concentration IPTG and the activity was measured under the standard conditions. (b) Effect of IPTG on the enzyme expression of the B0034+LeuDH. The enzymes were induced by different concentration IPTG and the activity was measured under the standard conditions. (c) Effect of IPTG on the enzyme expression of the B0032+LeuDH. The enzymes were induced by different concentration IPTG and the activity was measured under the standard conditions. (d) Effect of IPTG on the enzyme expression of the B0030+LeuDH. The enzymes were induced by different concentration IPTG and the activity was measured under the standard conditions. (e) Effect of IPTG on the enzyme expression of the 34L-34F (Enzyme activity of LeuDH, ●; Enzyme activity of FDH, □). The enzymes were induced by different concentration IPTG and the activity was measured under the standard conditions. (f) Effect of IPTG on the enzyme expression of the 32L-34F (Enzyme activity of LeuDH, ●; Enzyme activity of FDH, □). The enzymes were induced by different concentration IPTG and the activity was measured under the standard conditions. (g) Effect of IPTG on the enzyme expression of the 30L-34F (Enzyme activity of LeuDH, ●; Enzyme activity of FDH, □). The enzymes were

induced by different concentration IPTG and the activity was measured under the standard conditions. All experiments were performed three times, and each time had 2-3 parallel experiments. Error bars represent the standard deviation.

Fig.S4. HPLC detection of the L-*tert*-leucine and D-*tert*-leucine standards.

Fig.S5. Chromatogram of an artificially spiked sample containing 99.5% L-Tle and 0.5% D-Tle

Fig.S6. Chromatogram of enzymatic catalysis solution containing enzymatically synthesised product

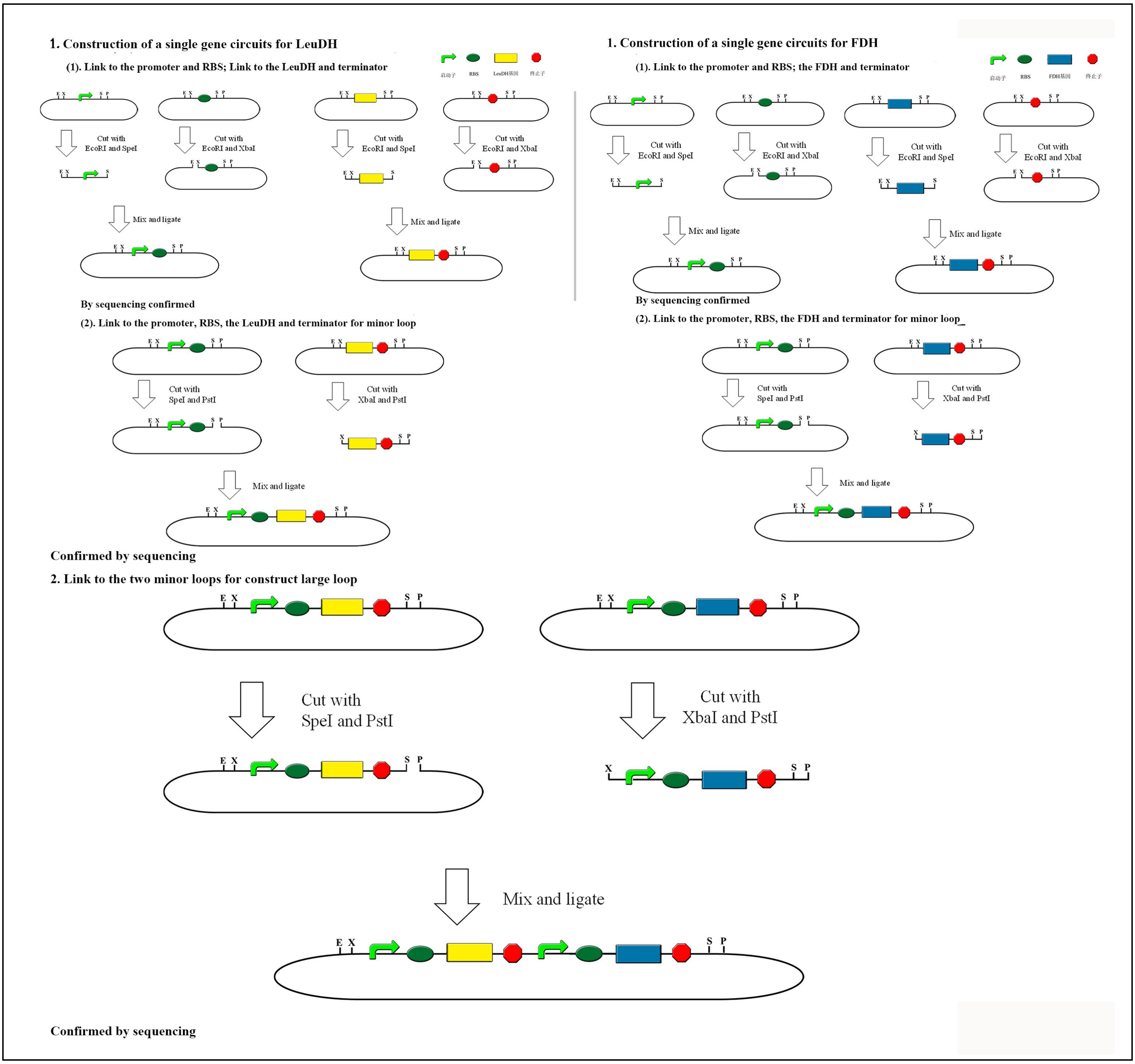

Figure S1 The methods and detail process of constructing the genetic circuits

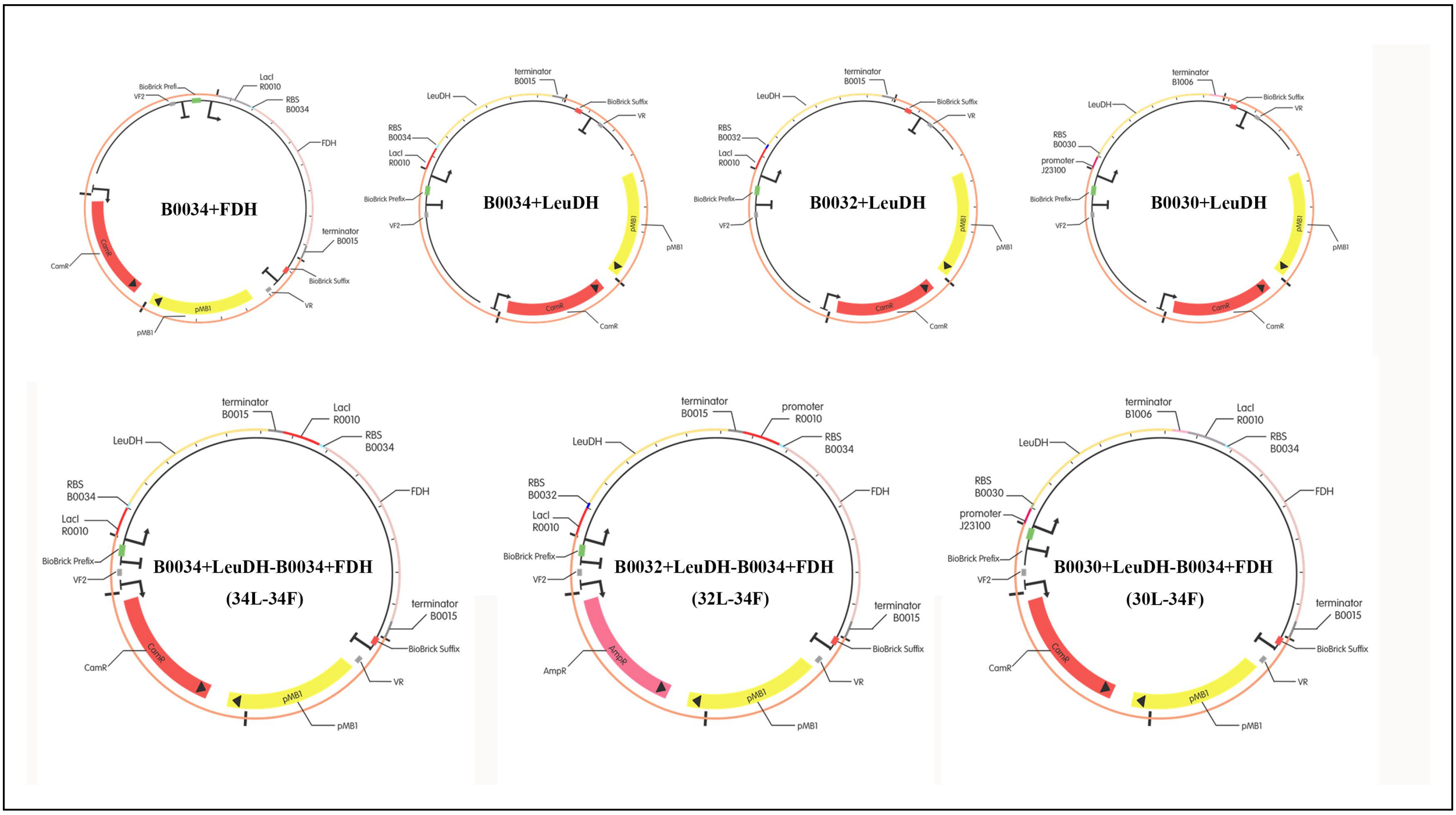

Figure S2 The physical maps of the genetic circuits

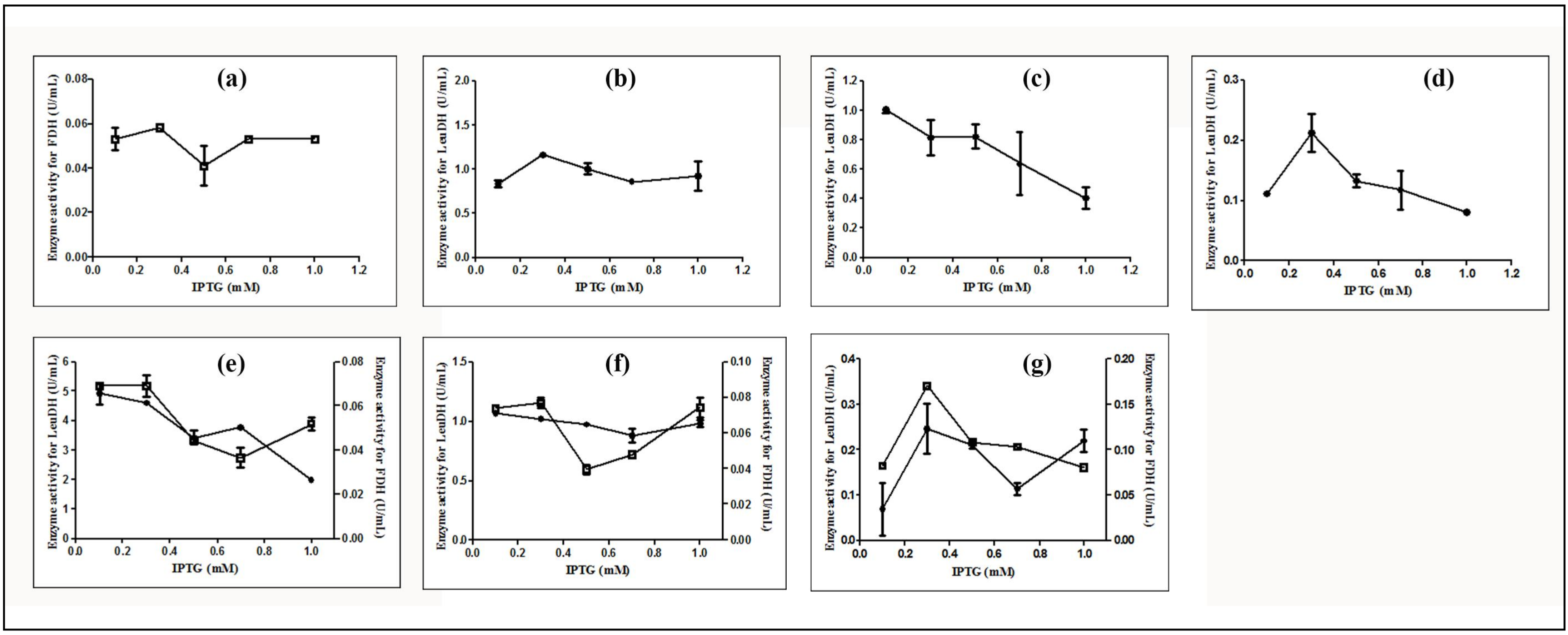

Figure S3 The suitable concentration of IPTG for expression of the genetic circuits

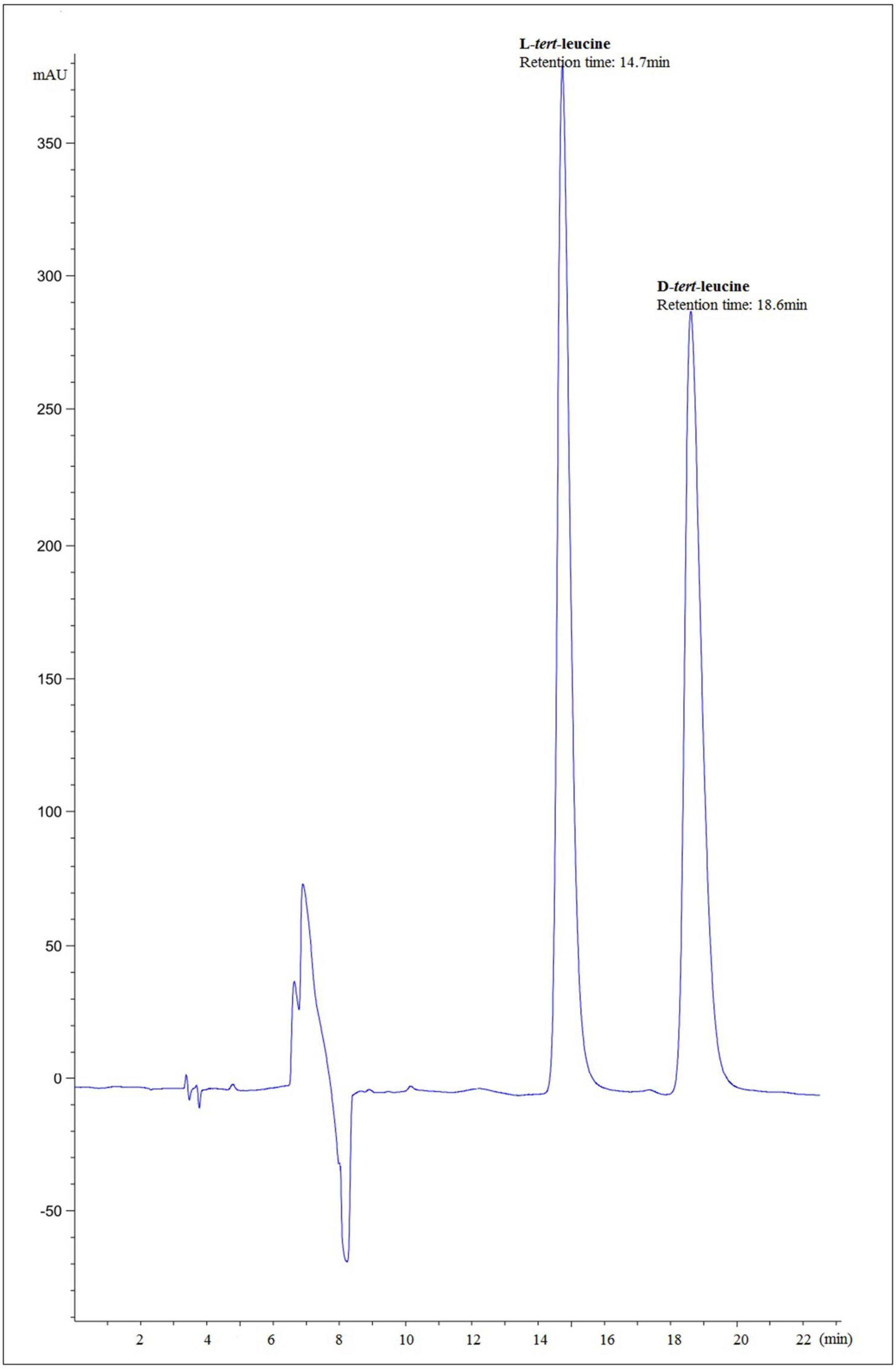

Figure S4 HPLC detection of the L-*tert*-leucine and D-*tert*-leucine standards

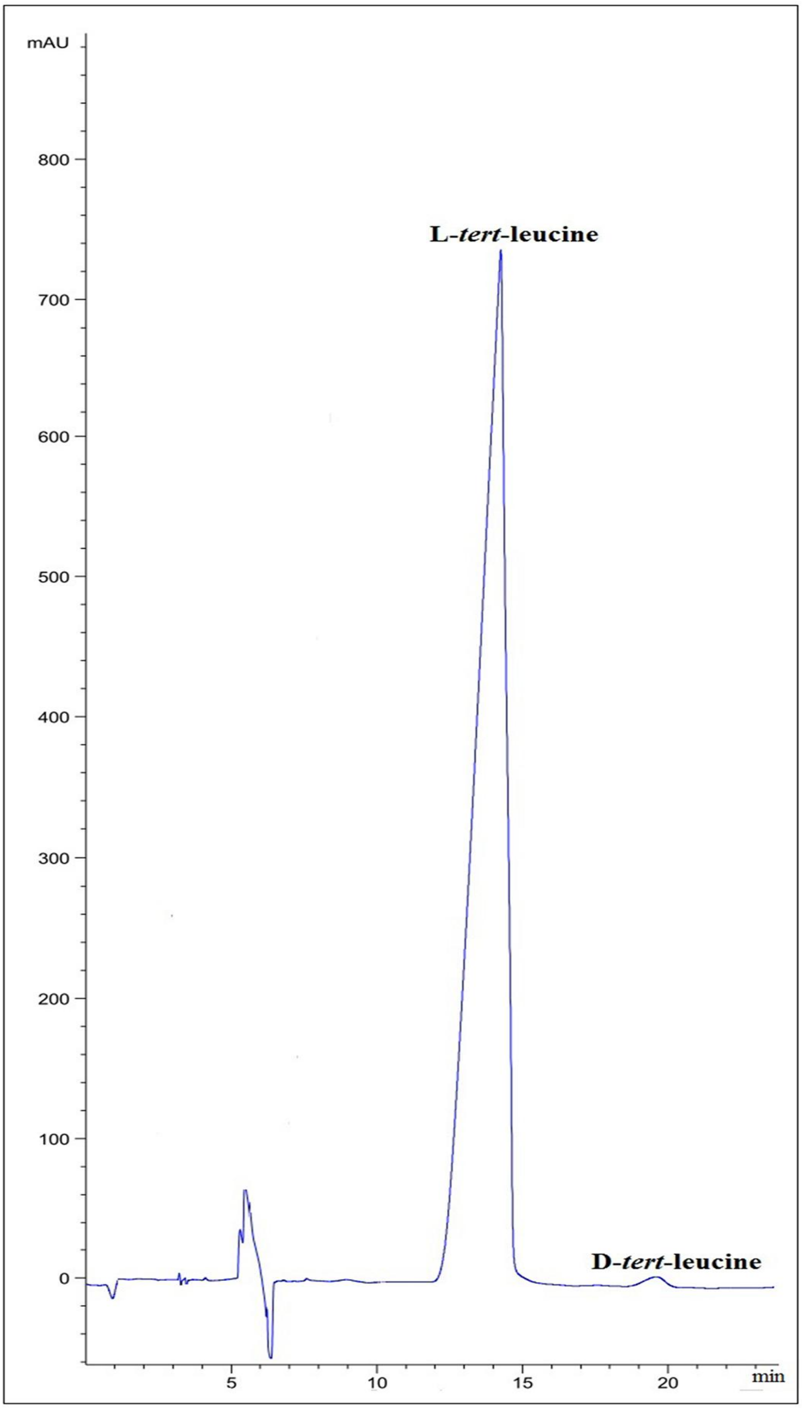

Figure S5 Chromatogram of an artificially spiked sample containing 99.5% L-Tle and 0.5% D-Tle

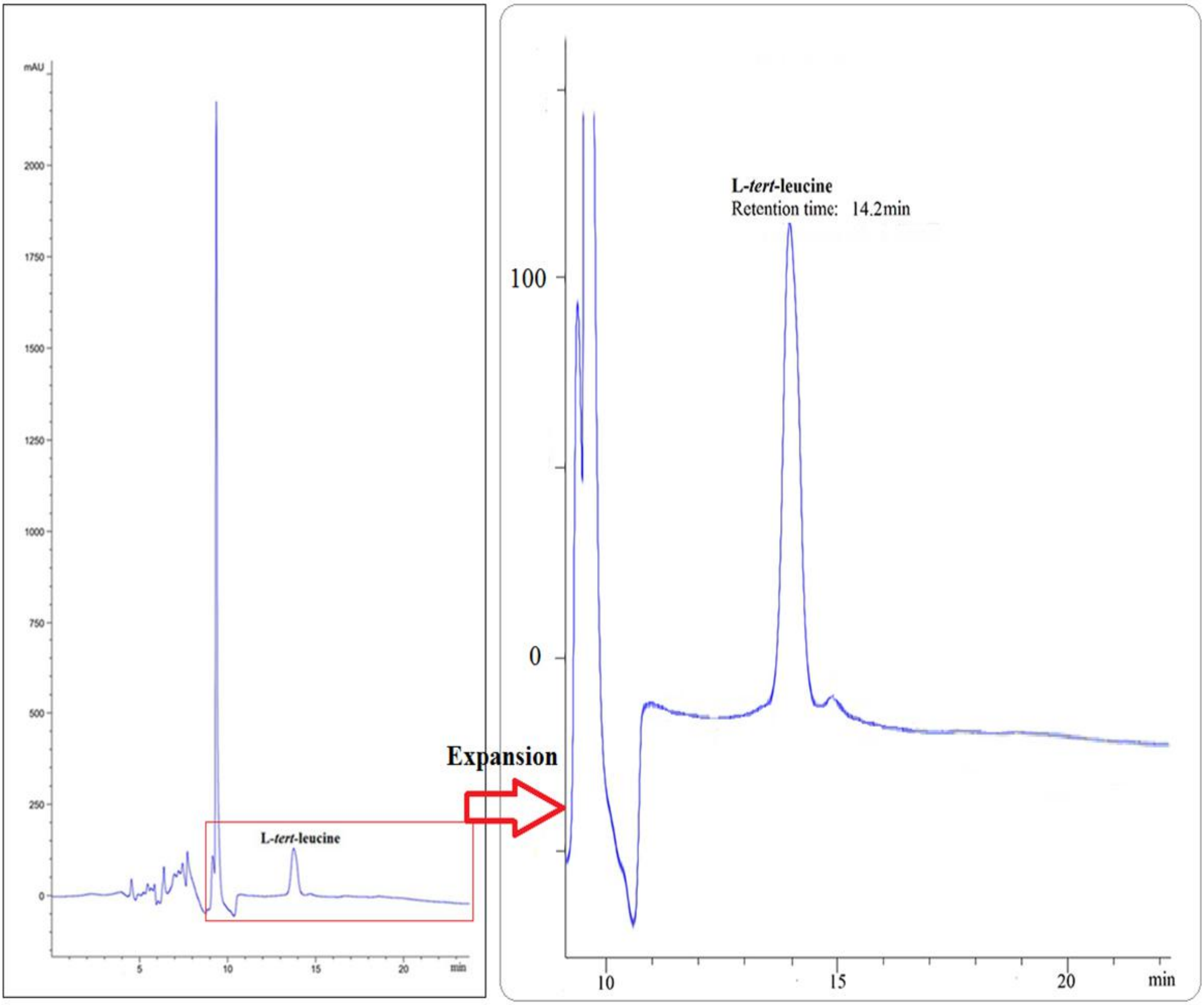

Figure S6 Chromatogram of enzymatic catalysis solution containing enzymatically synthesised product

Tables

Table 1 The sequence of the experimental primers

Primer	Sequences
LeuDH-F1	GTTTCTTCGAATTCGCGGCCGCTTCTAGATGACATTGGAAATCTTC
LeuDH-R1	GTTTCTTCCTGCAGCGGCCGCTACTAGTATTACCGGCGACTAAT
FDH-F1	GTTTCTTCGAATTCGCGGCCGCTTCTAGATGAAAATTGTCCTGG
FDH-R1	GTTTCTTCCTGCAGCGGCCGCTACTAGTATTACTTTTTATCGTGTT

Experimental section

Circuit construction

The plasmids pUC18-*leudh* (*leudh* expression cassette containing LeuDH gene from *B. cereus*) and pUC18-fdh (*fdh* expression cassette containing FDH gene from *C. boidinii*) synthesized by Sangon Biotech Ltd (Shanghai, China) were used as the template for PCR reaction with the primers LeuDH-F1, LeuDH-R1, FDH-F1 and FDH-R1, Table S1) in order to obtain the *leudh* and *fdh*. Our genetic circuits were constructed by using the standard biological parts from iGEM headquarter. The strength of the different RBS was determined by WABSAW (http://2010.igem.org/Team:Warsaw/Stage1/RBSMeas). The *leudh* and *fdh* was managed by double enzyme digestion with *EcoR*I and *Spe*I. After double digestion with *EcoR*I and *Spe*I, terminator B0015 was linked with each gene. Meanwhile, the RBS_B0034 and promoter LacI were linked with the same methods. After sequencing, the positive circuit psB1C3-*fdh*-terminator and psB1C3-*leudh*-terminator were digested by *Xba*I and *Pst*I. The circuit psB1C3-LacI-rbs_B0034 was digested by same restriction enzyme cutting sites. After ligation by T4 ligase enzyme, circuits made by promoter, rbs, gene and terminator are completed, generating B0034+LeuDH and B0034+FDH. After sequencing, the positive circuit B0034+LeuDH and B0034+FDH were digested by *Xba*I and *Pst*I. Then, there were legated by T4 ligase enzyme, the

B0034+LeuDH-B0034+FDH was constructed. All circuits at this stage were constructed with the same method. After that, another four circuits, B0032+LeuDH, B0030+LeuDH, B0032+LeuDH-B0034+FDH, B0030+LeuDH-B0034+FDH were constructed. Eventually, we obtained different circuits with *leudh* to the same circuits with *fdh*. The construction steps were shown in Figure S1.

Expression of the genetic circuits

The seven circuits, B0034+LeuDH, B0032+LeuDH, B0030+LeuDH, B0032+FDH, B0030+LeuDH-B0034+FDH, B0032+LeuDH-B0034+FDH and B0030+LeuDH-B0034+FDH were translated and *E. coil* BL21 (DE3) pLysS cells. The cells was cultivated at 37 °C until the optical density (OD600) reaching 0.5-0.8, the enzyme was induced with the different concentration of isopropylthiogalactoside (IPTG, 0.1Mm-1.0mM) at 18 °C for 12 h for determine the optimal induction conditions. Under the optimal induction condition, proteins were induced. Then, cells were harvested by centrifugation (8000×g, 10 min). The cell pellet was suspended with 7.5 ml of PBS buffer, and the cells were broken by Ultrasonic cell disruption. The broken cells were centrifuged (8000×g, 15min). Supernatant is the cell-free extract. The resulting enzyme was detected through enzyme activity and SDS-PAGE.

Enzyme and protein assays

Enzyme activity was detected by spectrophotometrically monitoring the reactions with the theory that the changes in the NADH concentration during the reactions result in changes in the absorbance at 340 nm (ε = 6220 M^{-1} cm^{-1}). The reductive amination reaction was started by adding NADH to the previously incubated reaction mixture. The oxidative deamination reaction was implemented by adding NAD^+ to the previously incubated reaction mixture. One unit (U) of enzyme activity was defined as the amount of enzyme catalyzing formation or degradation of 1 mol of NADH per min in the oxidative deamination or in the reductive amination under standard conditions.
